# Response to “The role of experience level in radiographic evaluation of femoroacetabular impingement and acetabular dysplasia”

**DOI:** 10.1093/jhps/hnv005

**Published:** 2015-03-13

**Authors:** Patrick Schottel, Caroline Park, Anthony Chang, Zak Knutson, Anil S. Ranawat

**Affiliations:** 1. Hospital for Special Surgery, New York, NY,; 2. Sharp Rees-Stealy Medical Center, San Diego, CA and; 3. Bone & Joint Hospital at St. Anthony, Norman, OK

We thank the authors for their valid and interesting viewpoints and we would like to address their questions.

The aim of the study was to emphasize the importance of properly performed and accurately interpreted radiographs for the proper diagnosis of femoroacetabular impingement (FAI) and acetabular dysplasia (AD). We believe that radiographs are the most critical imaging modality to help diagnose and treat patients with various hip disorders for all musculoskeletal physicians. Their importance are even greater now as healthcare costs have placed increased restrictions on the use of magnetic resonance imaging and computed tomography.

Although the author presents an interesting viewpoint, we do not believe that radiographic interpretation is more important at the level of the general radiologist or orthopaedic surgeon. We believe that radiographic interpretation is a fundamental skill that is critical for all physicians regardless of experience level. We incorporated a Sports Medicine Fellow as a surrogate for a general orthopaedic surgeon; however, radiographs play a critical role preoperatively, intra-operatively and postoperatively, thus accurate interpretation of radiographs is critical across varying experience levels, from generalists to specialists.

Furthermore, we do believe that the lower agreement found among observers using subjective assessments is relevant as a clinical tool in daily practice. Our study shows that using subjective criteria alone increases the susceptibility of over or under-reading radiographs, which readers should be aware of. Additionally, this places a greater emphasis on developing more objective criteria to improve the reliability of accurately diagnosing FAI and AD.

